# Lactosylated lipid calcium phosphate-based nanoparticles: A promising approach for efficient DNA delivery to hepatocytes

**DOI:** 10.22038/IJBMS.2024.76683.16602

**Published:** 2024

**Authors:** Masoomeh Khalifeh, Ali Badiee, Navid Ramezanian, Amirhossein Sahebkar, Atena Farahpour, Reza Kazemi Oskuee

**Affiliations:** 1 Department of Medical Biotechnology and Nanotechnology, Faculty of Medicine, Mashhad University of Medical Sciences, Mashhad, Iran; 2 Department of Pharmaceutical Nanotechnology, School of Pharmacy, Mashhad University of Medical Sciences, Mashhad, Iran; 3 Department of Chemistry, Faculty of Science, Ferdowsi University of Mashhad, Mashhad, Iran; 4 Applied Biomedical Research Center, Mashhad University of Medical Sciences, Mashhad, Iran; 5 Targeted Drug Delivery Research Center, Mashhad University of Medical Sciences, Mashhad, Iran

**Keywords:** Calcium Phosphates, DNA delivery, Hepatocytes, Nanoparticle, Polyethylenimine

## Abstract

**Objective(s):**

For safe and effective gene therapy, the ability to deliver the therapeutic nucleic acid to the target sites is crucial. In this study, lactosylated lipid phosphate calcium nanoparticles (lac-LCP) were developed for targeted delivery of pDNA to the hepatocyte cells. The lac-LCP formulation contained lactose-modified cholesterol (CHL), a ligand that binds to the asialoglycoprotein receptor (ASGR) expressed on hepatocytes, and polyethyleneimine (PEI) in the core.

**Materials and Methods:**

Fourier transform infrared spectroscopy (FT-IR) and nuclear magnetic resonance (NMR) were used to monitor the chemical modification, and the physicochemical properties of NPs were studied using dynamic light scattering (DLS) and transmission electron microscopy (TEM). To evaluate transfection efficiency, cellular uptake and GFP expression were assessed using fluorescence microscopy and flow cytometry.

**Results:**

The results revealed that lactose-targeted particles (lac-LCP) had a significant increase in cellular uptake by hepatocytes. The inclusion of a low molecular weight PEI (1.8 KDa) with a low PEI/pDNA ratio of 1 in the core of LCP, elicited high degrees of GFP protein expression (by 5 and 6-fold), which exhibited significantly higher efficiency than PEI 1.8 KDa and Lipofectamine.

**Conclusion:**

The successful functionalization and nuclear delivery of LCP NPs described here indicate its promise as an efficient delivery vector to hepatocyte nuclei.

## Introduction

In recent years, gene therapy has experienced significant progress, with the ability to target specific cells in various therapeutic tissues. However, effective delivery of nucleic acids *in vivo* remains challenging due to nucleolytic degradation in blood and rapid renal clearance. Furthermore, the physicochemical properties of nucleic acids, including high molecular weight (MW) and hydrophilic nature, prevent their penetration into the cell (1, 2). To overcome these obstacles, numerous delivery vectors have been fabricated to encapsulate genetic material during transport to the desired location. These vectors can be broadly divided into two families based on their origin: viral and non-viral. Despite the significant advantages of viral vectors in terms of delivery efficiency, their use is challenging due to the cost-effective commercial-scale production, immunogenicity, and limited loading capacity (3, 4). Therefore, various non-viral vectors have been devised over the years, including polymers, lipids, protein-based, organic, and inorganic particles (5). Among them, Lipid-based delivery systems, have been extensively studied and widely used for transferring various types of genetic material (6). 

The use of nucleic acid-based therapeutics for targeting the genetic background of hepatic disease has increased significantly in recent years (7). Since the hepatocytes constitute up to 80% of all liver cells and, owing to their diverse functions, they are potential targets for liver-related nucleic acid-based therapeutics (8). However, the liver sinusoidal epithelium represents a primary barrier between blood and liver parenchyma, with fenestration acting as a selective sieve that limits hepatocyte exposure (9, 10). Therefore, active targeting of hepatocytes via the asialoglycoprotein receptor (ASGPR) has been extensively explored (11). ASGPR is a lectin receptor highly expressed on hepatocytes, making them a desirable target for various glycans, such as N-acetylgalactosamine, galactose, and glucose (8). Among simple or complex sugars used as ligands, lactose has shown great affinity for ASGPR receptors, possibly due to galactose in their structure. Several lactosylated nanoparticles (NPs) have been synthesized to target hepatic tumors (12-14). 

After internalization into the target cell, the contents of the delivery vector must escape from the endosomes and reach their destination. While the site of action of RNAs is in the cytosol, DNA must penetrate the nuclei to access the proteins of the transcription machinery (15). Numerous strategies have been developed to augment the nuclear localization of non-viral vectors. These smart non-viral vectors have undergone modifications by incorporating peptides and other small molecules, which effectively facilitate the process of nuclear translocation (16, 17). Many previous studies have confirmed the role of polyethyleneimine (PEI) in the transport of PEI/DNA complexes through the cytoplasm toward the nucleus (18-20). PEI, as a non-viral cationic vector, effectively condenses plasmid DNA (pDNA) into nanostructure polyplexes through electrostatic interactions. These polyplexes can be taken up by the cells and further facilitate the transport of DNA to the cell nucleus (21). The research conducted by Sabtin *et al*. revealed that PEI can form channels in the membrane, enabling the transport of DNA into the cell nucleus by stabilizing the pores on the lipid membrane. Furthermore, the competition between the membrane and DNA for PEI binding is expected to facilitate the disassembly of polyplex and subsequent release of DNA (18). 

Huang *et al*., have recently developed lipid-coated calcium phosphate (LCP) NPs as a versatile platform for the systemic delivery of therapeutics, particularly genetic material. In LCP NPs, calcium phosphate (CaP) was coated with a bilayer lipid, resulting in improved colloidal stability of CaP, and the outer layer lipids have the potential for further modification and functionalization. Additionally, the CaP core of LCP NPs disassembles in acidic endosomes, resulting in the release of cargo into the cytoplasm (22, 23). To achieve efficient DNA delivery to hepatocytes, we synthesized lactose-modified cholesterol moieties (CHL) and incorporated them into the outer leaflet of LCP NPs to enhance specific interactions with receptors. Apart from functionalizing LCPs with lactose, we uniquely co-loaded the low molecular weight PEI (PEI 1.8 KDa) in the core of LCP to address the problem of endosomal escape and pDNA transit to the nucleus. 

## Materials and Methods

Lactose, Cholesterol, adipic acid, N, N0 -dicyclohexylcarbodiimide (DCC), 4-dimethylaminopyridine (DMAP), 1-Ethyl-3-(3-dimethyl aminopropyl) carbodiimide (EDC), igepal-CO520, calcium chloride, sodium acetate, DAPI (4′,6-diamidino-2-phenylindole), and MTT (3-(4,5-dimethyl-2-thiazolyl)-2,5-diphenyl-2Htetrazolium bromide) were purchased from Sigma-Aldrich Co. (MO, USA). Polyethyleneimine (PEI 1800 Da) was purchased from Polysciences Inc (Pennsylvania, USA). All other chemicals were obtained from Merck (Germany). All other lipids were purchased from Avanti Polar Lipids, Inc. (Alabaster, AL, USA). 1,1′-Dioctadecyl-3,3,3′,3′-tetramethylindocarbocyanine perchlorate (DiI) was obtained from Invitrogen (Carlsbad, CA, USA). Human hepatocellular carcinoma cells (Huh-7) were obtained from the Pasteur Institute (Iran). Roswell Park Memorial Institute (RPMI-1640) medium, penicillin-streptomycin, and trypsin were purchased from Betacell (Iran), and Fetal Bovine Serum (FBS) was purchased from GIBCO (Gaithersburg, USA). 

***Synthesis of cholesterol-modified lactose (CHL)***

This synthesis was performed in two steps: Initially, Adipic acid (4.2 g), cholesterol (1 g), and DMAP (19.3 mg) were dissolved in 35 ml of anhydrous THF under argon. Then, the dissolved DCC (1.5 g) in THF (5 ml) was added dropwise to the mixture in an ice-water bath. The reaction mixture was stirred for 16 hr at room temperature under argon. The product was filtered, and the solvent was removed using rotary evaporation. The intermediate product was purified by recrystallization from a mixture of n-hexane and ethyl acetate. The resulting precipitate was cholesterol-adipic acid monoesters (CH-AA).

**Scheme 1:**
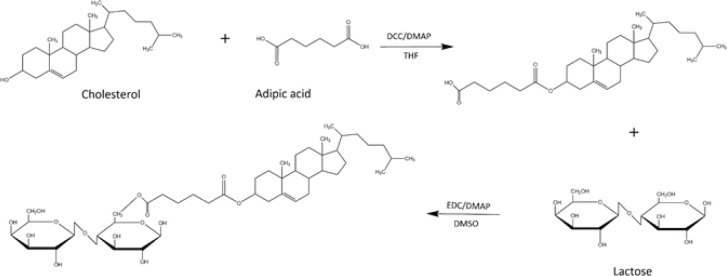
Synthesis of cholesterol-modified lactose (CHL)

At the next step, CH-AA in dried DMSO was activated using DMAP (1/100 equivalent) and EDC (1/5 equivalent). Activated CH-AA (1/3 equivalent lactose) was added to the lactose solution in DMSO. The mixture was stirred at 40 °C for 16 hr. Then, the solution was dried with a freeze-dryer, and the pellet was washed with absolute ethanol. Finally, the precipitate was dried under a vacuum to obtain CHL conjugates. The final product contains cholesterol attached to adipic acid and lactose (Scheme 1). The chemical structure of CHL was confirmed via FT-IR (KBr pellets) and 1 H-NMR and 13C- NMR using CDCL3 and DMSO-d6 as solvents.

***Plasmid extraction and purification***

The eGFP plasmid was propagated in the host of Escherichia coli DH5α. Plasmids were extracted using the EndoFree plasmid Giga kit (Qiagen, Hilden, Germany). The pDNA concentration was determined by measuring the absorbance at A260. pDNA was dissolved (1 mg/ml) in sterile PBS and stored at −20 °C.

***Preparation and characterization of lactose-decorated LCP NPs loaded with pDNA***

LCP-based NPs were prepared as previously described with a slight modification (24). The anionic lipid-coated CaP core was formed in a water-in-oil microemulsion and further coated with the second lipid layer. Briefly, two separate microemulsions (5 ml) of Igepal-520 and cyclohexane (3: 7 v: v) were prepared and placed under stirring. To form the Ca phase, 20 µg of pDNA was added to 200 µl of 2.5 M CaCl_2_. Meanwhile, the phosphate phase was prepared by dispersing 200 µl of 12.5 mM Na_2_HPO_4 _(pH 9) and DOPA (70 µl, 20 mM) into a separate 5 ml oil phase. Two emulsions were stirred for 10 min and then mixed and stirred for an additional 20 min. An equal volume of absolute ethanol was added to break the microemulsion, and the mixture was centrifuged at 10,000 g for 20 min to collect the CaP cores. After that, the pellet was washed twice with ethanol to completely remove surfactants and cyclohexane. The precipitate was dissolved in chloroform for further use. The final LCP NPs were produced by mixing CaP cores with 180 μl of 20 mM DOTAP/cholesterol/ DSPE-PEG (molar ratio = 55/25/20). For lactose-decorated LCP NPs, the lipid mixture of the outer leaflet was DOTAP/lac-Cholesterol/ DSPE-PEG (molar ratio = 60/30/20). After desiccation of chloroform, the residual lipids were rehydrated in deionized water. The morphology of CaP cores was characterized using transmission electron microscopy (TEM) (JEOL 100CX II TEM, JEOL, Japan). Zeta potential and particle size of LCPs were measured using a Zetasizer system (Zetasizer nano zs, Malvern Instruments Ltd., Worcestershire, UK). 

***Preparation of PEI/pDNA polyplex and LCP/PEI/pDNA lipopolylpex***

PEI/pDNA polyplexes were prepared in HBG buffer by adding PEI-1800 solution to pDNA solution at an N/P ratio of 2 and further incubated at room temperature for 20 min. Lipopolyplexs were obtained by adding PEI 1.8 (N/P ratio 1) to the Ca phase solution before mixing the two microemulsions, followed by the same method of LCP preparation. 

***Cell culture***

Huh-7 cells (Human hepatoma cell line) were maintained in RPMI 1640 medium supplemented with 10% FBS, penicillin (100 IU/ ml), and streptomycin (100 mg/ ml) at 37 °C in a humidified atmosphere with 5% CO_2_.

***Cellular uptake***

Fluorescent microscopy and flow cytometry were employed to examine cellular uptake. DiI-labeled LCP NPs were prepared by adding 2% DiI to the lipids. Huh-7 cells were seeded in a 24-well cell culture plate at a density of 2 × 10^5^ cells per well and maintained for 24 hr at 37 °C, 5% CO2. The medium was then replaced with 1 ml of medium containing LCP NPs (1 µg of DNA per well). After four hours of incubation with NPs, the medium was changed and the cells were fixed for 10 min in 4% paraformaldehyde, followed by three washes with PBS before obtaining the fluorescent microscopy images. The coverslips containing the cells were then mounted on a glass slide with 3 μl of DAPI, and fluorescent microscopy was used to acquire the images. The quantitative cellular uptake of LCP NPs was further evaluated via flow cytometry. After 4 hours of incubation, the culture media was removed, and the cells were washed three times with PBS, trypsinized, and collected by centrifugation. The cell pellets were redispersed in 0.3 ml PBS and the fluorescent intensity of the cells was determined using flow cytometry (BD FACSCaliburTM, BD Biosciences, San Jose, USA). The fluorescence of 10,000 single cells was measured for each sample. 

***Transfection experiments with eGFP pDNA***

Huh-7 cells were seeded into a 24-well plate at 2 × 10^5^ cells/well. After 24 hours of incubation, cells were treated with various NPs loaded with eGFP pDNA (1 µg of DNA per well). After 4 hours of incubation, the medium was replaced with fresh medium and incubated for 48 hr. After the incubation, the cells were washed twice with 1x PBS, detached using 0.05% trypsin-EDTA, and then centrifuged at 1000 rpm for 5 min. The cell pellets were resuspended in 0.3 ml PBS by gentle pipetting and were analyzed using flow cytometry (BD FACSCaliburTM, BD Biosciences, San Jose, USA). 

***Statistical analysis***

The results were expressed as the mean value with standard deviation (mean ± SD). The unpaired t-test was used to compare the mean between two groups, and one-way ANOVA was used to compare more than two groups. A difference with a *P*-value less than 0.05 (*P*<0.05) was considered statistically significant. Data was analyzed with GraphPad Prism 8.0 software. 

## Results

***Synthesis and characterization of CHL***

**Figure 1:**
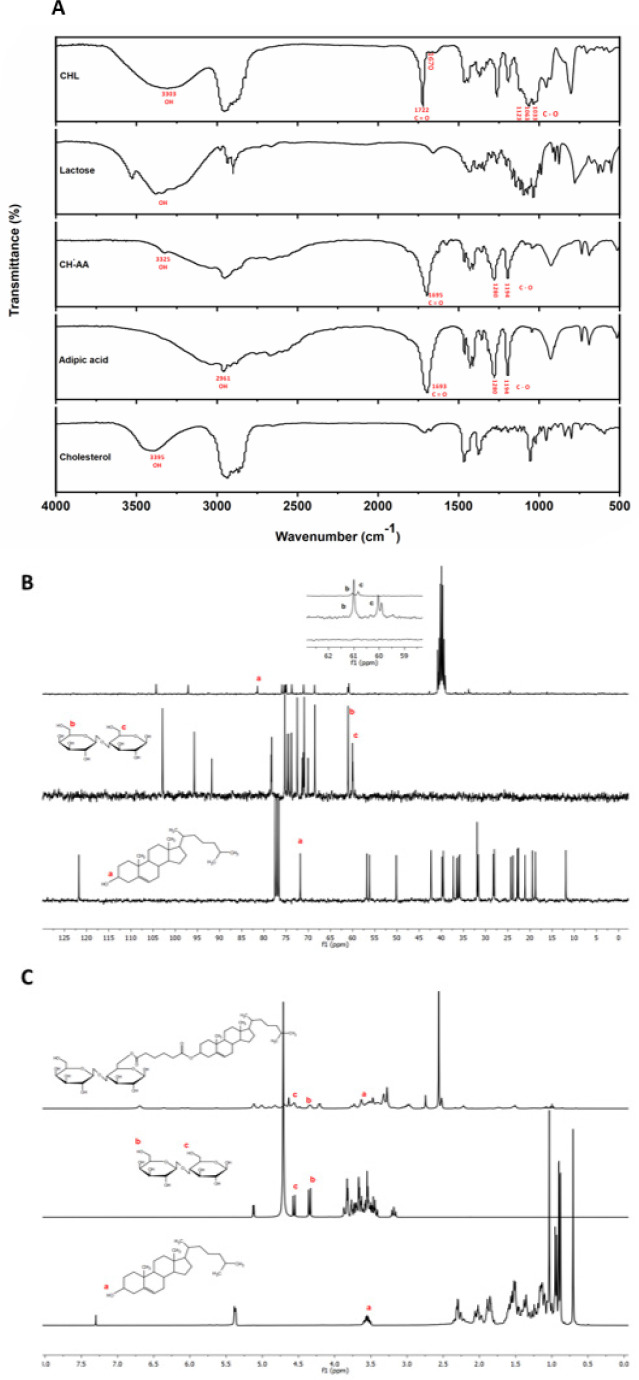
(A) FT-IR spectrum of cholesterol, adipic acid, cholesterol-adipic acid (CH-AA), lactose, and cholesterol-modified lactose (CHL). (B) 13C-NMR and (C) 1H-NMR of cholesterol, lactose and CHL

To increase the specificity and affinity of LCP NPs to ASGPR on hepatic cells, we synthesized a ligand for the ASGPR receptor by conjugating cholesterol to a lactose moiety. The designed conjugate was prepared via a two-step reaction, as shown in Scheme 1. In the first step, to attach cholesterol to lactose, cholesterol was reacted with adipic acid to produce CH-AA through an ester linkage between the hydroxyl group of cholesterol and the carboxyl group of adipic acid. In the second step, CH-AA was bonded to lactose by forming an ester bond between the carboxyl group of CH-AA and the hydroxymethyl group of lactose. The carboxyl of the intermediate product, CH-AA, was activated by adding EDC and DMAP as catalysts. The structure of the compounds confirmed by ^13^C-NMR, ^1^ H-NMR, and FT-IR are shown in Figure 1. 

The infrared spectra of cholesterol, adipic acid, CH-AA, lactose, and CHL are displayed in Figure 1A. The carbonyl stretch of the acid in adipic acid and CH-AA was characterized by bands at 1693 cm^-1^ and 1695 cm^-1^, respectively. The presence of a peak at 1722 cm^-1^ is assigned to the carbonyl stretch of the ester group in CHL. Aliphatic CH stretching vibrations for CH-AA were observed at 2951 cm^-1^ and for CHL at 2956 cm^-1^ and 2868 cm^-1^, which can be assigned to the presence of cholesterol function in the structures. A broad band at 3303 cm^-1^ in the CHL spectra is attributed to the OH stretch of various hydroxyl groups in lactose. The results of FT-IR analysis preliminarily showed that the desired compound was synthesized according to the proposed pathway outlined in Scheme 1. 

We also performed proton and carbon NMR analyses of cholesterol, lactose, and CHL. The disappearance of the hydroxyl proton present in cholesterol at 3.55 ppm, as shown in Figure 1 C, indicated the formation of an ester bond. Furthermore, peaks attributed to lactose hydroxymethyl groups (a and b) were observed in CHL. In carbon NMR (Figure 1B), the peak at 71.8 ppm corresponding to carbon (a) of cholestene shifted to 81.45 ppm in CHL. Additionally, absorption peaks at 61.007 ppm and 60.041 ppm were attributed to the hydroxymethyl groups (b, c) in lactose, which shifted to 61.07 ppm and 60.83 ppm in CHL. These findings suggest that CH-AA was coupled with lactose mainly by hydroxymethyl groups, confirming the presence of lactose in CHL. 

***Characterization of LCP and LCP-PEI NPs***

To enhance the efficiency of pDNA delivery by LCP NPs to hepatic cells, lactose was conjugated to cholesterol through a two-step reaction. The surface of LCP NPs was functionalized by adding CHL to the outer leaflet lipids, allowing the formation of the outer leaflet with the resulting glycolipid into the outer lipid layer. 

**Figure 2:**
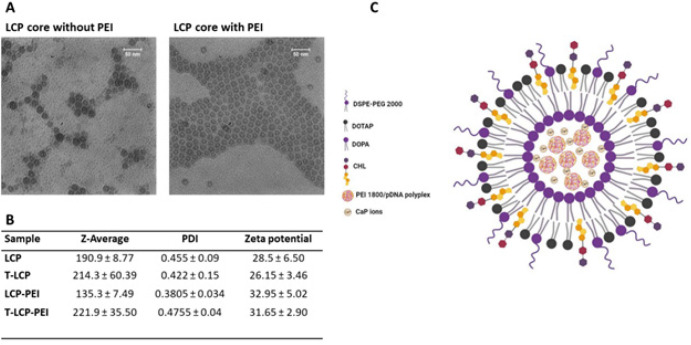
Characterization of lactose-targeted LCP (lac-LCP) NP

The physicochemical and morphological properties of pDNA-loaded LCP NPs were characterized by dynamic light scattering (DLS) and TEM techniques. The TEM image showed that the core of LCP NP has a spherical hollow structure with a diameter of approximately 25 nm (Figure 2A). The average hydrodynamic diameter and surface charge of the LCP and lac-LCP NPs are outlined in Figure 2B. The surface charges were positive and were narrowly dispersed around 30 mV. The diameter of the lac-LCP NP was slightly higher than the nontargeted LCP NP. 

Adding PEI (1.8 KDa) to the core of LCP NPs may facilitate encapsulation by interacting with phosphate residues of DNA and increasing the coprecipitation of DNA with CaP. TEM images obtained from LCP cores containing PEI (Figure 2A) showed that they were well-dispersed spherical vehicles, indicating that the pDNA molecules were sufficiently condensed by PEI and CaP. The average particle sizes of LCP-PEI and lac-LCP-PEI prepared at a PEI: DNA ratio of 1 were 135.3 ± 7.4 nm and 221.9 ± 35.5 (Figure 2B), respectively. The zeta potential of LCP-PEI was higher than that of the corresponding LCP (figure 2B), possibly due to adding the positive charge of PEI to the LCP core. Representative features of lac-LCP-PEI nanoparticles are shown in Figure 2C. 

***Cellular uptake of lactose-targeted LCP (lac-LCP) NPs***

**Figure 3:**
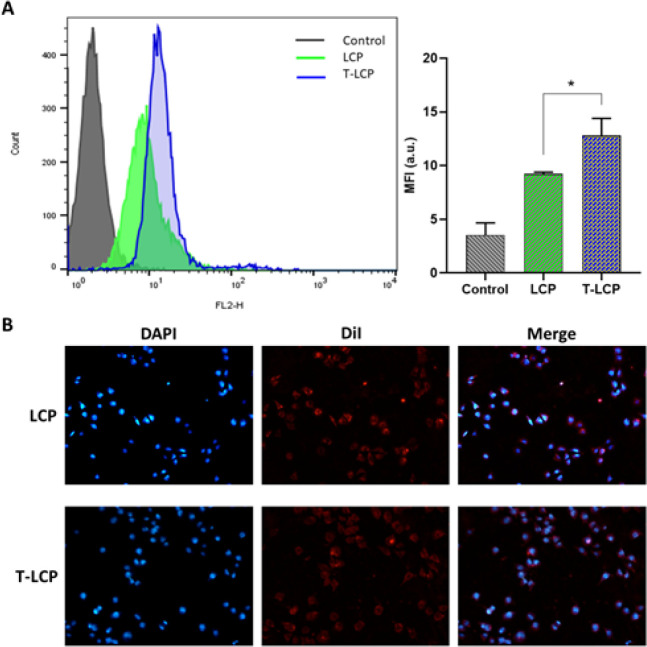
Cellular uptake of DiI-labeled LCP NPs

To analyze the cellular uptake of lac-LCP NPs, flow cytometry was employed. Huh-7 cells, a human hepatoma cell line that overexpresses the ASGPR receptor, were used as they are an appropriate cell line for studying the uptake of lac-LCP NPs. The cellular uptake was performed by incorporating DiI dye in the lipid layer of LCPs. Huh-7 cells were incubated with LCP formulations for 4 hr, and the mean fluorescence intensity of (MFI) of cells that internalized DiI-labeled NPs was determined using flow cytometry analysis (Figure 3A). Lactose-free LCPs were used as non-targeted controls. As illustrated in Figure 3, lac-LCP NPs exhibited a higher MFI (1.4 fold) than non-targeted LCP, demonstrating that the addition of ASGPR-targeting ligand significantly enhanced cellular uptake by Huh-7 cells. Furthermore, Huh-7 cells were incubated with lac-LCP and LCP solutions and observed under a fluorescence microscope. In Figure 3B, Huh-7 cells treated with both LCP and lac-LCP exhibited red fluorescence, however, the cells treated with lac-LCP showed stronger internal fluorescence.

***Transfection studies with pDNA***

**Figure 4:**
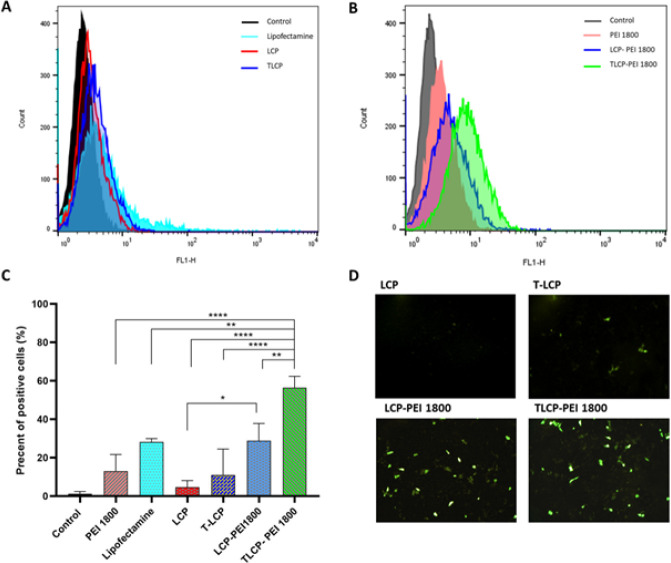
Gene expression ability in transfected cells

At first, pDNA was encapsulated in LCP NPs to evaluate the transfection efficiency for pDNA delivery, and Lipofectamine 3000 was used as a positive control. After 48 hr of transfection, GFP protein expression was measured using flow cytometry analysis. As depicted in Figure 4A, treatment with LCP and lac-LCP did not result in gene expression. This indicates that LCP NPs successfully entered the cells but failed to translocate to the nucleus. To address this issue, we co-encapsulated PEI (1.8 KDa) at a PEI/pDNA ratio of 1 to enhance pDNA delivery to the hepatocyte nuclei. The results demonstrated (Figures 4A and B) that by combining LCP with PEI, the GFP expression significantly increased compared with the LCP and lac-LCP without PEI. Given that the nuclear trafficking of DNA is an essential requirement for the expression of pDNA (25), it is supposed that the enhanced effectiveness of the LCP-PEI and lac-LCP-PEI can be attributed to increased intracellular pDNA trafficking. Additionally, fluorescence microscopy was employed to visualize the GFP expression in the cells (Figure 4D), revealing that the inclusion of PEI polymer in the core LCP NPs improved DNA delivery at a post-endosomal level. 

(a: characteristic chemical shift of 3´-methine of cholesterol, b,c: characteristic chemical shifts of hydroxymethyl of lactose).

(A) Morphology of LCP cores loaded with pDNA or pDNA/PEI by TEM imaging. (B) Size distribution and zeta potential for LCP and lac-LCP, loaded with pDNA and/or pDNA/PEI. (C) Illustration of membrane core LCP NPs with asymmetric lipid membrane functionalized with lactose ligand

Huh-7 cells were treated with lac-LCP and LCP for 4 hr, and the fluorescent signals were determined by (A) flow cytometry and (B) fluorescent microscopy.

(A) Huh-7 cells treated with LCP NPs loaded with pDNA or (B) pDNA/PEI for 4 hr, with the fluorescent intensity measured after 48 hr. (C) A comparison of GFP protein expression in Huh-7 cells treated with LCP, T-LCP, and Lipofectamine 3000 NPs. (D) Fluorescent microscopy images of GFP expression in cells after incubation with formulations with or without PEI. Magnification 10 x.

## Discussion

The liver is an attractive target for developing gene therapy strategies due to its crucial role in various metabolic activities, the inherent liver tropism of many gene therapy vehicles, and its ability to produce proteins for distribution throughout the body. In recent years, there has been a remarkable advance in the toolbox available for targeted gene delivery to the liver. Successful gene therapy would require a suitable delivery system with selectivity (4). Many efforts have been made to achieve liver cell-specific drug delivery for liver disease treatment. Among the various receptors on the surface of parenchyma cells, the ASGPR receptor is an attractive target due to its exclusive expression as well as rapid internalization and high affinity for the ligand (6, 26). Carbohydrate-receptor interaction offers potential advantages including high avidity, low toxicity, and exclusive expression (27, 28).

 In this study, we constructed new LCP NPs to enhance the efficiency of pDNA delivery to hepatocytes. LCP NPs serve as multifunctional gene vectors, which overcome the barriers to successful gene delivery by encapsulating nucleic acid into a pH-sensitive CaP core surrounded by a lipid bilayer (29). Lactose was used as targeting moieties because of its specific interaction with ASGPR receptors overexpressed on the hepatocytes (30, 31). Many studies have reported the development of lactosylated polymers or liposomes as drug/gene carriers to target the hepatocytes (12, 13, 32, 33). Esterification and amidation are the most common reactions in these conjugations, which occur through the coupling of hydroxyl or amino groups of carbohydrates and carboxylic acid groups of the lipid anchor (34-36). Here, we used cholesterol as a lipid anchor because cholesterol is one of the components of LCP and provides rigidity and stability. In addition, the acyl derivative of cholesterol can facilitate the chemical reaction (34). Lactose moieties were chemically bound to the cholesterol via an ester bond by adipic acid linker, allowing them to remain on the outer surface of the LCP NPs and effectively serve as a targeting moiety. FT-IR spectra confirmed the presence of an ester functional group at 1722 cm^-1^. Also, the ^1^H-NMR spectrum of the product showed the absence or change of signals at 3.5 ppm, 4.33 ppm, and 4.56 ppm, corresponding to hydroxyl and hydroxymethyl groups in cholesterol and lactose (a, b, c). These observations suggest that these groups were involved in the reaction. 

Flow cytometry results demonstrated selective internalization of lactose-containing LCPs into Huh-7 cells through ASGPR receptor mediation, in contrast to non-specific binding observed with LCP alone. This finding is consistent with previous studies (12, 13) and supports the notion that lactose-containing NPs have the ability to target liver cells. For non-targeted LCP NPs, endocytose in cells occurs through electrostatic attraction between LCP and cell membranes. 

After demonstrating specific targeting of lac-LCP NPs to Huh-7 cells, further *in vitro* studies were conducted to examine GFP gene expression. Despite the strong cellular uptake, lac-LCP and LCP showed low gene expression. While LCP NPs have a high pH buffering capacity due to the cationic lipids and pH-sensitive CaP core, which improve the endosomal release (23), effective nuclear delivery remains a significant challenge in establishing efficacy. According to the above results, LCP NPs showed a weak ability to deliver pDNA to the nucleus that can arise from the endosomal release or pDNA transfer to the nucleus. According to the study by Hu *et al*., which focused on pDNA delivery to hepatocytes, galactose-targeted LCPs were taken up by the majority of hepatocytes in the liver. However, Cy3-labeled pDNA was predominantly distributed in the cytoplasm. To improve the intracellular delivery of pDNA to the nucleus, they encapsulated octaarginine in LCP nanoparticles (15) to mimic the nuclear localization signal (NLS) (15). To address this challenge, we constructed lipopolyplexes with LCP and PEI by loading the PEI polymer with pDNA in the core of LCP NPs. PEI, as a non-viral vector, acts as a “proton sponge” at low pH, facilitating endosomal escape and influencing the cytoplasmic transfer of pDNA, directing it to the nucleus (21, 37). We used low MW PEI, PEI 1.8 KDa, for coprecipitation with pDNA and CaP in the core of LCPs. The MW of PEI plays a crucial role in polyplex formation and those with lower MW typically show poorer DNA condensation. However, this tendency is diminished when polyplexes are enclosed within liposomes due to the unique properties of the lipid membrane (38, 39). Therefore, shorter chains of PEI are mainly employed for lipopolyplex synthesis owing to their low cytotoxicity (40, 41). There was a slight difference in physicochemical properties between formulations with and without PEI. The lipopolyplexes containing PEI showed higher zeta potentials than LCPs without PEI, which is probably due to the positive charge of PEI. The LCP containing PEI showed significantly higher gene expression than LCP formulations without PEI. GFP signals indicated that the carriers had the ability to release pDNA from the lysosomes and subsequently transport it to the nucleus. Furthermore, the transfection efficacy increased in ligand-mediated active targeting via the lac-LCP-PEI carrier. 

## Conclusion

In this work, we successfully developed a gene delivery vehicle consisting of a lipid-coated CaP core carrying the pDNA and PEI in the core, along with a targeting lactose moiety. The lactose-targeting ligand was synthesized by conjugating lactose with cholesterol (CHL) and incorporated into the LCP structure. Lac-LCP-PEI NPs with the nanoscale size, spherical structure, and positive charge showed improved cellular uptake and more effective pDNA delivery to the nucleus of Huh-7 cells. Furthermore, the incorporation of PEI into the LCP cores improved gene expression. These lac-LCP-PEI NPs serve as a powerful platform with the potential to overcome the contradiction between cellular uptake capacity and nuclear localization. This work provides an example of how new targeted nanocarriers for DNA delivery can be constructed.

## Authors’ Contributions

R KO, A B, N R, and A S contributed to the conception and design of the research. M K contributed to the implementation of the research, writing the manuscript, and analysis of the results. A F contributed to data acquisitions. 

## Conflicts of Interest

The authors declare that no conflict of interest exists.
